# Identification of microRNAs associated with human fragile X syndrome using next-generation sequencing

**DOI:** 10.1038/s41598-022-08916-4

**Published:** 2022-03-23

**Authors:** Maryam Sotoudeh Anvari, Hamed Vasei, Hossein Najmabadi, Reza Shervin Badv, Akram Golipour, Samira Mohammadi-Yeganeh, Saeede Salehi, Mahmood Mohamadi, Hamidreza Goodarzynejad, Seyed Javad Mowla

**Affiliations:** 1 Department of Molecular Pathology, School of Medicine, Children’s Medical Center, Pediatrics Center of Excellence, Tehran University of Medical Sciences, Tehran, Iran; 2 Department of Mathematical Science, Sharif University of Technology, Tehran, Iran; 3 Department of Genetics, School of Rehabilitation Sciences, Genetic Research Center, The University of Social Welfare and Rehabilitation Sciences, Tehran, Iran; 4 Department of Pediatrics, School of Medicine, Children’s Medical Center, Pediatrics Center of Excellence, Tehran University of Medical Sciences, Tehran, Iran; 5 Department of Biology, Science and Research Branch, Islamic Azad University, Tehran, Iran; 6 Medical Nanotechnology and Tissue Engineering Research Center, Shahid Beheshti University of Medical Sciences, Tehran, Iran; 7 Department of Medical Biotechnology, School of Advanced Technologies in Medicine, Shahid Beheshti University of Medical Sciences, Tehran, Iran; 8 Cell-Based Therapies Research Center, Digestive Diseases Research Institute, Shariati Hospital, Tehran University of Medical Sciences, Tehran, Iran; 9 Department of Basic and Clinical Research, Tehran Heart Center, Tehran University of Medical Sciences, Tehran, Iran; 10 Department of Molecular Genetics, Faculty of Biological Science, Tarbiat Modares University, Tehran, Iran

**Keywords:** Genetics, Molecular biology, Molecular medicine, Neurology

## Abstract

Fragile X syndrome (FXS) is caused by a mutation in the *FMR1* gene which can lead to a loss or shortage of the *FMR1* protein. This protein interacts with specific miRNAs and can cause a range of neurological disorders. Therefore, miRNAs could act as a novel class of biomarkers for common CNS diseases. This study aimed to test this theory by exploring the expression profiles of various miRNAs in Iranian using deep sequencing-based technologies and validating the miRNAs affecting the expression of the *FMR1* gene. Blood samples were taken from 15 patients with FXS (9 males, 6 females) and 12 controls. 25 miRNAs were differentially expressed in individuals with FXS compared to controls. Levels of 9 miRNAs were found to be significantly changed (3 upregulated and 6 downregulated). In Patients, the levels of hsa-miR-532-5p, hsa-miR-652-3p and hsa-miR-4797-3p were significantly upregulated while levels of hsa-miR-191-5p, hsa-miR-181-5p, hsa-miR-26a-5p, hsa-miR-30e-5p, hsa-miR-186-5p, and hsa-miR-4797-5p exhibited significant downregulation; and these dysregulations were confirmed by RT‐qPCR. This study presents among the first evidence of altered miRNA expression in blood samples from patients with FXS, which could be used for diagnostic, prognostic, and treatment purposes. Larger studies are required to confirm these preliminary results.

## Introduction

Fragile X syndrome (FXS, OMIM 300624), a neurodevelopmental disorder characterized by intellectual disability and autism, is caused by a mutation in the fragile X mental retardation 1 (*FMR1*) gene located on the X chromosome at Xq27.3 wherein a DNA segment, known as the CGG triplet is expanded within the 5′ untranslated region (5′ UTR) of *FMR1* gene^1^.

Regarding American College of Medical Genetics (ACMG) in FXS, CGG triplet is repeated more than 200 times (that is known as a “full” mutation); those with a premutation and intermediate genotype carry a 55–200 and 45–54 of CGG repeats respectively, while this DNA segment is normally repeated between 5 and 44 times^2^. The carriers of premutation show a much lesser extent FXS phenotype^3^, but are at risk of developing a neurodegenerative disorder in adulthood in male, called fragile X tremor-ataxia syndrome (FXTAS) and Fragile X-associated primary ovarian insufficiency, or FXPOI among female carriers^4, 5^.

Full mutations cause the *FMR1* genes to “turn off” resulting in shutting down its ability to produce a functional protein, fragile X mental retardation 1 protein (FMRP). FMRP is an RNA binding protein acting as a translational repressor of a variety of messenger RNA (mRNA) targets at the synapse that, though present in many tissues, is thought to play a pivotal role in synaptic maturation, functioning of neurons, and their communication^6^. Therefore, its loss or shortage leads to FXS and induces a range of neurodevelopmental problems that include cognitive impairment, learning disabilities, hyperactivity^7^, seizures, sleep problems, especially aggressive behavior^8^ autism spectrum disorder (ASD), and anxiety^9^.

This protein is associate not only with specific mRNAs and with microRNAs (miRNAs) but also with the components of the miRNA pathway such as the Dicer and Argonaute proteins^10^. The miRNAs, small (~ 22-nucleotide) single-strand noncoding RNAs, negatively regulate target mRNA expression or activity of genes; they act as a guide by base-pairing with target mRNA. The type of silencing mechanism employed, i.e., translation inhibition or cleavage of target mRNA with subsequent degradation is determined by the level of complementarity between the guide and mRNA target^11, 12^. Many miRNAs are involved in neurodevelopmental disorders. Neural communication impairment as a result of miRNA disruption, perhaps contributes to the development of FXS as the primary cause of inherited intellectual disability^13^.

Hence, unsurprisingly, in many neuropsychiatric disorders, it has been demonstrated that the dysregulation of miRNAs is associated with changes in behavior, learning, and memory^14^. Understanding the miRNA-mediated translational regulation mechanism(s) whereby FMRP modulates the translation of its mRNA ligands would help in the understanding of the molecular pathogenesis of FXS and also of converging mechanisms shared by FXS and its related disorders^15^. FXS is the most common known cause of autism spectrum disorder (ASD) and inherited intellectual disability^8, 9, 16^; list of other related disorders to FXS may include but not limited to FXTAS, Rett syndrome, Down syndrome, attention deficit hyperactivity disorder (ADHD), and schizophrenia^17^.

There are twelve brain miRNAs identified to interact with FMRP in mouse brain including, miR-125a, miR-125b, and miR-132^18^. To the best of our knowledge, there is only one published study on miRNA expression profiling in patients with FXS using deep sequencing^19^. In a twin study for identifying miRNA biomarkers of this disorder, the levels of miRNAs in the urine of a boy with FXS and his twin brother, who was a premutation carrier and had no clinical signs of fragile X, were compared^19^. Using next-generation sequencing, the investigators found twenty-eight miRNAs with different levels between the two twins. In the brother with fragile X, eight miRNAs had higher levels, the greatest increase (i.e., a 1.6-fold increase) was found in the levels of miR-125a compared to his twin brother^19^. This increase in miR‐125a levels was also shown in two other sets of urine samples; ten Spanish FXS children aged 2–7 years and nine Finnish FXS children aged 4–17 years, as compared to healthy subjects but with a wide variation in urine miR-125a levels among young fragile X patients^19^.

miRNAs could serve as a novel class of potential biomarkers for the diagnosis and prognosis of common central nervous system (CNS) diseases including, neurodevelopmental disorders, and offer novel therapeutics^20–22^. The present study aimed to explore the expression profiles of various miRNAs in patients with FXS using deep sequencing-based technologies and validate the miRNAs that affect the expression of *FMR1*gene in a set of blood samples among an Iranian population.

## Methods and materials

### Study participants

Between November 2016 and June 2019, a total of 27 individuals, some of whom are related, were recruited for this study from several different cities across Iran. For this study, we recruited fifteen patients with different types of FXS (full mutation, premutation and full mutation mosaics) from four families in three generations. Diagnoses were confirmed via southern blot analysis at the University of Social Welfare and Rehabilitation Sciences (USWR), and the patients included nine males (between 25 and 36 years old) and six females (between 48 and 80 years old).

Twelve sex- and age-matched healthy individuals were taken as controls. All participants provided written informed consent, in agreement with the Declaration of Helsinki for research involving human subjects, explicitly gave permission for RNA analyses and gathering the relevant clinical data. The National Institute for Medical Research Development (NIMAD) ethics committee approved the study protocol and use of human blood for research (No. 957806). Participants were asked to fill out a questionnaire detailing their family history of FXS, associated disease, food habits, and medication history for the evaluation of miR interactions. Data regarding the result of food habits and medication history were not included in the analyses due to concerns about the accuracy of these data.

Autism spectrum disorder, Attention deficit hyperactivity disorder and seizure were diagnosed by neurology team using available diagnostic tools at different times. All mentioned patients, were known cases of ASD,ADHD and seizure in the past and were receiving or completing treatment.

### Specimen collection and DNA/RNA extraction

Each participant provided 10–15 ml cubital vein blood samples that were placed in ethylenediamine tetra-acetic acid (EDTA)-containing (lavender top) tubes. The genomic DNA was isolated from peripheral blood leukocytes using QIAamp DNA Blood Mini Kit (Qiagen, Hilden, Germany), according to the manufacturer's instructions by using the spin column extraction method. Erythrocytes were lysed in Erythrocyte-Lysis-Buffer (Buffer EL, Qiagen) and total RNA including small RNAs, was extracted using miRNeasy Mini Kit (Qiagen, Hilden, Germany) as per the manufacturer's protocol.

### CGG repeat primed PCR

Molecular analysis of the *FMR1*gene CGG repeat locus was performed in all participants to reconfirm diagnosis and to provide accurate sizing of alleles. To assess the number of CGG repeats in the 5′ UTR of the *FMR1*, a three-primer CGG Repeat Primed PCR [RP-PCR] was carried out on the purified genomic DNA. Samples were PCR-amplified by an Eppendorf Mastercycler gradient PCR system (Eppendorf, Hamburg, Germany) using the AmplideX PCR/CE *FMR1* Kit (Asuragen, Austin, TX) and AmplideX PCR/CE*FMR1*reagents (cat. no. 49402), following the manufacturer’s instructions. PCR products were then separated in a 3310XL capillary electrophoresis system [Applied Biosystems Genetic Analyzer, Foster City, CA, USA] based on conditions described in the kit manual. GeneMapper software (ID-X Software Version 1.0), Applied Biosystems, was used to analyze and convert the separated PCR products into CGG repeat length using. In accordance with the current ACMG Guidelines, the CGG triplets that repeated 45–54 and 55–200 times were considered as intermediate and premutation respectively. Those with more than 200 repeats were defined as full mutation. Samples with both premutation and full mutation alleles were identified as full mutation mosaics.

### Total RNA quantity and quality control

All isolated RNA samples were eluted in RNase‐free water and RNA concentrations were determined with Nanodrop Spectrophotometer (Thermo Scientific, Wilmington, DE, USA). RNA quantity was evaluated by calculating absorbance at λ = 260 nm, and the quality was assessed by a ratio of λ = 260/280 nm being close to 2.0–2.3. The RNA concentration of each sample was more than 50 ng. The integrity of the RNA, as a key feature that affects the performance of sequencing and RT-qPCR, was assessed via two methods: First, by running extracted RNA through 1% agarose gel and then staining with ethidium bromide to observe the 28S ribosomal RNA band at 4.5 kb, and the 18S rRNA band at 1.9 kb. Second, by using the Agilent 4200 TapeStation System (Agilent Technologies, Santa Clara, CA, G2991A) to assess the electrophoretic profile of the 18S and 28S RNA and generating an RNA Integrity Number (RIN). All RNA samples revealed RIN values of greater than eight and the miRNA extractions were stored at − 80 °C until processing.

### Library preparation for next-generation sequencing

The TruSeq Small RNA Library Preparation Kit (Illumina, San Diego, USA) was used for generating miRNA sequencing libraries directly from total RNA as per the manufacturer's protocol for this kit (TruSeq Small RNA Library Prep Reference Guide 15004197 v02). Briefly, after ligation of the 5′ and 3′ RNA adapters using T4 RNA ligase, reverse transcription was performed to generate cDNA; cDNA libraries were subsequently amplified by PCR. The products were then purified. After acrylamide gel purification, eight libraries were pooled in an equimolar amount to create one lane and validation was compatible with multiplexed sequencing. Finally, the libraries were checked and normalized according to protocol.

### miRNA profiling through next-generation sequencing (miRNA-seq)

The miRNA cDNA libraries were sequenced on an Illumina MiniSeq platform in the Pars gene company, Shiraz, Iran. With this platform, DNA fragments of the libraries go through clonal amplification by bridge PCR followed by sequencing using a reversible terminator. It consists of sequencing by synthesis (SBS) technology using only two-channel (i.e., red and green) which needs only two images for determination of all four base calls reducing the number of cycles, cost, and time required for data processing and yet delivering high accuracy and quality^23^. Within every cycle of sequencing, for each cluster, base calls were created by inbuilt real-time analysis software and raw data were stored in the format of individual base call files (*.bcl). The BCL files were converted to standard FASTQ file formats (a text-based sequencing data file format that stores both raw sequence data and quality scores) for downstream analysis.

FASTQ data acquisition in available repositories, Sequence Read Archive (SRA: Accession number: PRJNA777620) in NCBI was obtained and is in processing to transfer to GEO.

In the next step, the output data were streamed into Illumina’s BaseSpace Sequence Hub for cloud-based data management and analysis. FASTQ files were cleaned by adapter removal using CutAdapt 1.6. The Phred numerical quality scoring system was used as a base call quality filter^24, 25^; reads with Q scores of < 33 (checked both before and after adapter trimming) were removed. After removal, the adaptors and filtering out low-quality sequences, the processed reads were aligned against the miRBase database and human genome hg19 by using version 2.2.3 of Short Read Mapping Package (SHRiMP). The number of reads mapped to miRNAs in each sample provided as SHRiMP log table and trimming-mapping plot are illustrated in supplementary 1, 2 respectively. According to the SHRiMP log table, the range of Reads is 519045 to 2456946 and the percentage of mapped reads for all miRNAs is 2.79–83.67% as minimum and maximum. Mapped files were then sorted and indexed as binary format (BAM) files.

### Relative quantification of miRNA by reverse-transcription PCR analyses

Quantitative reverse transcription-polymerase chain reaction (qRT-PCR) is a well-established method for miRNA profiling with the highest sensitivity and accuracy and with the widest dynamic range^26^. We used stem-loop RT-PCR miRNA assay for quantification of miRNA expression levels^27^ using a commercially available qRT-PCR miRNA detection kit and primer sets (Zist Fanavari Pishgam Company, Tehran, Iran). To normalize the expression data, a commonly-used endogenous reference gene, U6 small nuclear RNA (U6-snRNA), was adopted^28^. Real-time PCR data with the use of a LightCycler 96 instrument (Roche Applied Science, Indianapolis, IN, USA), were analyzed by 2^−ΔΔCT^ method using the following equation: ΔΔCT = ((Ct miRNA of concern − Ct U6-snRNA) in patients) − ((Ct miRNA of concern − Ct U6-snRNA) in controls)^29^. These relative gene expression data analyses were double-checked by using the relative expression software (REST-2009) tool^30^.

The MicroRNAs gene expression was compared between sexes and number of repeats (genotypes) and was analyzed using independent sample t and ANOVA tests (Table 5).

To evaluate the degree of similarity between RT-qPCR and miRNA-seq results, we compared miRNAs’ expression data generated through both approaches. Downregulation was defined when 2^−ΔΔCT^ was less than zero and upregulation presented as more than zero results of 2^−ΔΔCT^.

### Differentially expressed miRNA analysis of deep sequencing data

To find differentially expressed miRNAs between case and controls, we have to compare expression levels of nearly 1800 miRNAs that have read count numbers greater than zero for at least one of the two groups. This leads to 1800 statistical tests. When multiple hypotheses are tested simultaneously, an adjustment for the multiplicity of tests is often necessary to restrict the total number of false discoveries, and usage of such methods has become standard in genomics^31^.

The most conservative method for adjusting *p* values is Bonferroni correction^31, 32^. There exist other adjustment methods which increase the power of the statistical test in the cost of decreasing control over type I error. Our study using Bonferroni correction leads to over a hundred candidate miRNAs but we just want to choose a handful number of them for further investigations, so statistical power is not an issue here.

A reasonable way of choosing miRNAs for further exploration would be choosing miRNAs with the lowest *p* values. However, because the lowest *p* values vary significantly by adding or removing some samples, they may be too sensitive to noise. Also, their differences are too small and their ordering may not be robust too. So we used a slightly different approach other than simply picking miRNAs with the lowest *p* values. In this study, to determine differentially expressed miRNAs, we conducted differential expression analysis using four different software tools, namely, DESeq2 version1.26.0 (https://bioconductor.org/packages/release/bioc/html/DESeq2.html), edgeR version 3.28.1 (https://bioconductor.org/packages/release/bioc/html/edgeR.html), limma-trend and limma-voom version 3.42.2 (https://bioconductor.org/packages/release/bioc/html/limma.html). We listed 30 miRNAs with the lowest *p* values in each method and computed intersections of these 30 candidates. Interestingly, the agreement among the methods in calling differentially expressed miRNAs was high, despite their differences in assumptions and algorithms and also in the order and magnitude of their resulting *p* values. The overall intersection consists of 25 miRNAs which showed noteworthy agreement between different methods (Table2).

## Result

### CGG allele sizing and clinical characteristics of studied subjects

The clinical and demographic findings of the studied patients are listed in Table 1. The specific genotype category results are characterized as full mutation (n = 10), premutation (n = 3), and full mutation mosaic (n = 2). In this study, all (100%) available full mutation and full mutation mosaic patients with FXS presented with ASD, and one-third (33%) had ADHD.

**Table 1:** Clinical characteristics of studied subjects.

Family no	Case no	Relationship	Gender	Age/year	CGG repeats	Phenotype	Genotype	ADHD	Seizure	Autism
1	1	Grand mother	Female	80	68.66/68.66	Normal looking	Premutation	N	N	N
	2	Daughter	Female	48	312/30.2	Affected	Full mutation	N	P	P
	3	Son	Male	44	311.8	Affected	Full mutation	P	P	P
	4	Daughter (mother of cases 5 and 6)	Female	54	104.4/104.4	Normal looking	Premutation	N	N	N
	5	Grand son	Male	25	310.8	Affected	Full mutation	P	N	P
	6	Grand son	Male	27	310.8	Affected	Full mutation	P	P	P
2	7	Son	Male	36	312.36	Affected	Full mutation	Not available	Not available	Not available
	8	Son	Male	34	319.4	Affected	Full mutation	Not available	Not available	Not available
	9	Mother of cases7 and 8	Female	62	318/22	Normal looking	Full mutation mosaic	Not available	Not available	Not available
3	10	Son	Male	29	317	Affected	Full mutation	P	N	P
	11	Son	Male	32	318	Affected	Full mutation	N	N	P
	12	Mother of cases10 and 11	Female	57	88/22	Normal looking	Premutation	N	N	N
4	13	Son	Male	28	316	Affected	Full mutation	P	N	P
	14	Son	Male	26	316	affected	Full mutation	N	N	P
	15	Mother of cases 13and 14	Female	59	227.2/30	Normal looking	Full mutation mosaic	N	N	P

*ADHD* attention deficit hyperactivity disorder, *P* positive, *N* negative.

### Differentially expressed deep sequencing of miRNA

As explained in “Differentially expressed miRNA analysis of deep sequencing data” section, we first picked 25 miRNA in (Table2), utilizing four differential expression analysis tools DESeq2, edgeR, limma-trend, and limma-voom. To apply further investigations on the miRNAs we needed to filter this list to obtain only 9 miRNAs. The number of chosen miRNAs depends on what type of further exploration we are going to apply and its cost.

**Table 2:** Twenty-five proposed miRs in deep sequencing using four differential expression analyses.

miR no	miR name	Regulation mode in case samples	Overall mean expression	Case mean expression	Control mean expression	Log fold change
1	hsa-miR-191-5p	Down	8876.373051	1276.631887	18,376.0495	3.940923422
2	hsa-miR-26a-5p	Down	7813.136539	1442.80566	15,776.05014	3.487235906
3	hsa-miR-181a-5p	Down	6185.741177	1183.864533	12,438.08698	3.702044447
4	hsa-miR-30e-5p	Down	3199.723878	357.0752659	6753.034643	4.141018947
5	hsa-miR-186-5p	Down	1701.390164	559.7558769	3128.433024	2.572602978
6	hsa-miR-532-5p	Up	442.5755252	711.2084902	106.7843188	− 2.843199798
7	hsa-miR-652-3p	Up	167.1577007	258.3405092	53.17919022	− 2.189509984
8	hsa-miR-4797-3p	Up	101.9033055	176.4535552	8.715493351	− 3.969467534
9	hsa-miR-4797-5p	Up	101.3007798	175.4015655	8.674797652	− 3.96298818
10	hsa-miR-139-5p	Up	68.4866255	114.8116871	10.58029852	− 3.162100671
11	hsa-miR-210-3p	Up	57.60665272	96.72746231	8.705640726	− 3.349322696
12	hsa-miR-339-5p	Up	54.82137012	86.19747425	15.60123996	− 2.301431168
13	hsa-miR-548av-5p	Down	23.67638935	2.193939122	50.52945214	3.943386072
14	hsa-miR-548k	Down	23.65788696	2.193939122	50.48782175	3.942268815
15	hsa-miR-324-5p	Up	20.9344738	33.52838669	5.192082674	− 2.508217082
16	hsa-miR-148a-5p	Down	20.2027045	4.392828836	39.96504909	3.084674792
17	hsa-miR-148b-5p	Down	20.13664435	3.12648513	41.39934337	3.425508366
18	hsa-miR-24-2-5p	Down	15.03578544	5.367524883	27.12111113	2.4804667
19	hsa-miR-4485-3p	Up	14.65427465	24.97665627	1.751297614	− 3.515081463
20	hsa-miR-30d-3p	Down	8.531824974	2.279761135	16.34690477	2.904832494
21	hsa-miR-548av-3p	Down	8.264467723	1.266091684	17.01243777	3.266046604
22	hsa-miR-141-3p	Down	8.06856032	0.45925296	17.58019452	3.776080468
23	hsa-miR-548f.-5p	Down	7.741525273	0.151715543	17.22878743	4.027732978
24	hsa-miR-548o-3p	Down	7.364951245	0.930963985	15.40743532	3.212444121
25	hsa-miR-559	Down	7.351270372	0.773720351	15.5732079	3.335571389

These 25 miRNAs cannot be sorted based on *p* values because each of the DE analysis tools used, imposes a different *p* value ordering on this list. From the perspective of fold changes, the absolute value of fold changes for all the resulted miRNAs was over 2 with a majority of them over 3, which is not surprising, because fold change is already somehow taken into account when computing *p* values. Hence we did not use *p* value or fold change as the parameter for this round of filtering.

We sorted the list of these 25 miRNAs based on the overall mean expression level for all cases and controls and chose the first nine highly expressed miRNAs. The intuition behind this selection was that we expected these miRNAs to present more powerful signals and lower noises as opposed to miRNAs with a lower level of expressions and we expect their *p* values to be more robust.

miR-125, though was not in 30 highly dysregulated candidate miRNAs in this study, was also analyzed due to its importance in urine in the Putkonen et al.^19^ study and the existence of a small number of NGS studies about FXS. As seen in Table 3, all members of the miR-125 family were found to be downregulated in patients with FXS.

**Table 3:** hsa-miR-125 family in deep sequencing using four different methods for differential expression analysis.

	Mir	U/D	LFC	Limma-Trend	LFC	Limma-Voom	LFC	edgeR	LFC	DESeq2
1	125a-5p	Down	2.27	4.66E−06	2.3	2.72E−06	1.97	2.67E−06	1.54	0.00030171
2	125b-2-3p	Down	0.6	2.42E−05	0.82	0.0540679	3.81	2.60E−06	1.9	0.01564258
3	125a-3p	Down	0.4	0.00922126	0.3	0.43746489	1.43	0.01400016	1.01	0.13573104
4	125b-5p	Down	0.31	0.29037969	0.29	0.4493121	0.2	0.58630045	− 1.27	0.95661198

*U/D* up /down, *LFC* log fold change.

### Proposed miR reconfirmation by RT-PCR

**Figure 1:**
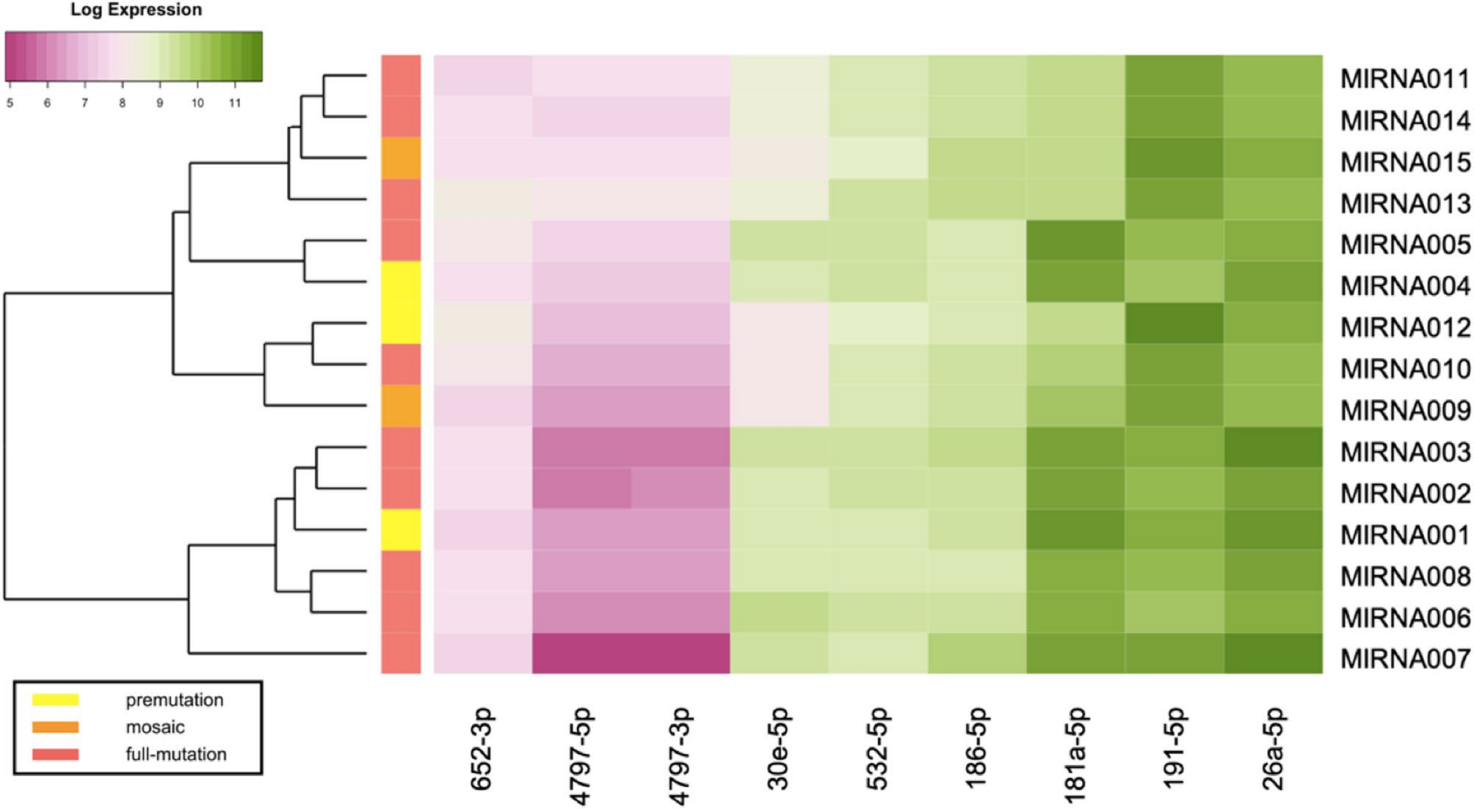
Log Expression-related Heat map and hierarchical clustering of miRNA based on three groups of full mutations, permutation, full mutation mosaic. Each row represents a case, and each column represents a miRNA.

The schematic heat map shows log expression-related changes in miRNA transcriptome based on three groups of full mutations, permutation, full mutation mosaic (Fig. 1).

Data regarding the most expressed miRNAs in ten full mutation samples are given in Table 4. hsa-miR-181a-5p downregulated in 90% of patients and the highest upregulated miRs were hsa-miR-4797-3p and hsa-miR-652-3p. Through RT-PCR, a significant downregulation of miR-181a-5p, miR-26a-5p, and miR-30e-5p, as well as miR-191-5p and miR-186a, was observed in all premutation patients (n = 3), while miR-652-3p was upregulated in all three premutation patients. In two cases identified as full mutation mosaics, five humans (Homo sapiens) miRNAs, i.e., miR-181a-5p, miR-26a-5p, miR-532-5p, miR-191-5p, and miR-4797-5p, were revealed to be downregulated, but no common upregulated miRNA was found.

**Table 4:** List of differentially expressed miRNAs among the full mutation group.

MicroRNA	Dysregulation	Percent of affected patients (%)
hsa-miR-181a-5p	Downregulated	90
hsa-miR-26a-5p	Downregulated	80
hsa-miR-4797-5p	Downregulated	70
hsa-miR-186-5p	Downregulated	60
hsa-miR-191-5p	Downregulated	60
hsa-miR-4797-3p	Upregulated	50
hsa-miR-652-3p	Upregulated	50
hsa-miR-30e-5p	Up/Down*	40/40

*40% of patients expressed downregulation and 40% upregulation.

Regarding the difference between the expression of miRs in the three groups of premutation, full mutation, and mosaic, only miR-532-5p has a significantly different distribution in the three groups. Using ANOVA, the most overexpression was found in full-mutation and then premutation and mosaic respectively (*p* value = 0.0423, Df = 2, F-value = 4.165).

There was no difference between men and women in genes expression of miRs (*p* vlaue > 0.05). Some miRNAs including miR-191-5p, miR-26a-5p, and miR-181-5p were downregulated, whereas miR-652-3p was upregulated in all six females. These miRNAs seemed to be gender-dependent. Similarly, age dependency was seen in those aged over 55 years with miR-191-5p, miR-26a-5p, and miR-181-5p. Among These miRs, expression of miR-532-5p gene was significantly more in patient under 55 years than over 55 years (*p* value = 0.03416, df = 4.5305, t = 2.9979).

In addition, no significant correlation has existed between any miRNAs and ADHD (*p* value > 0.05). The presence of seizure disorder was documented in three patients that all showed downregulation of miR-26a-5p and miR-186-5p. Autism spectrum disorder was significantly present in full mutation patients with downregulated miR-181-5p (*p* value < 0.05). In search in targetscan7.7 database, (http://www.targetscan.org/vert_72/) for prediction of miRNA target, no target was found for miR-181-5p.

**Figure 2:**
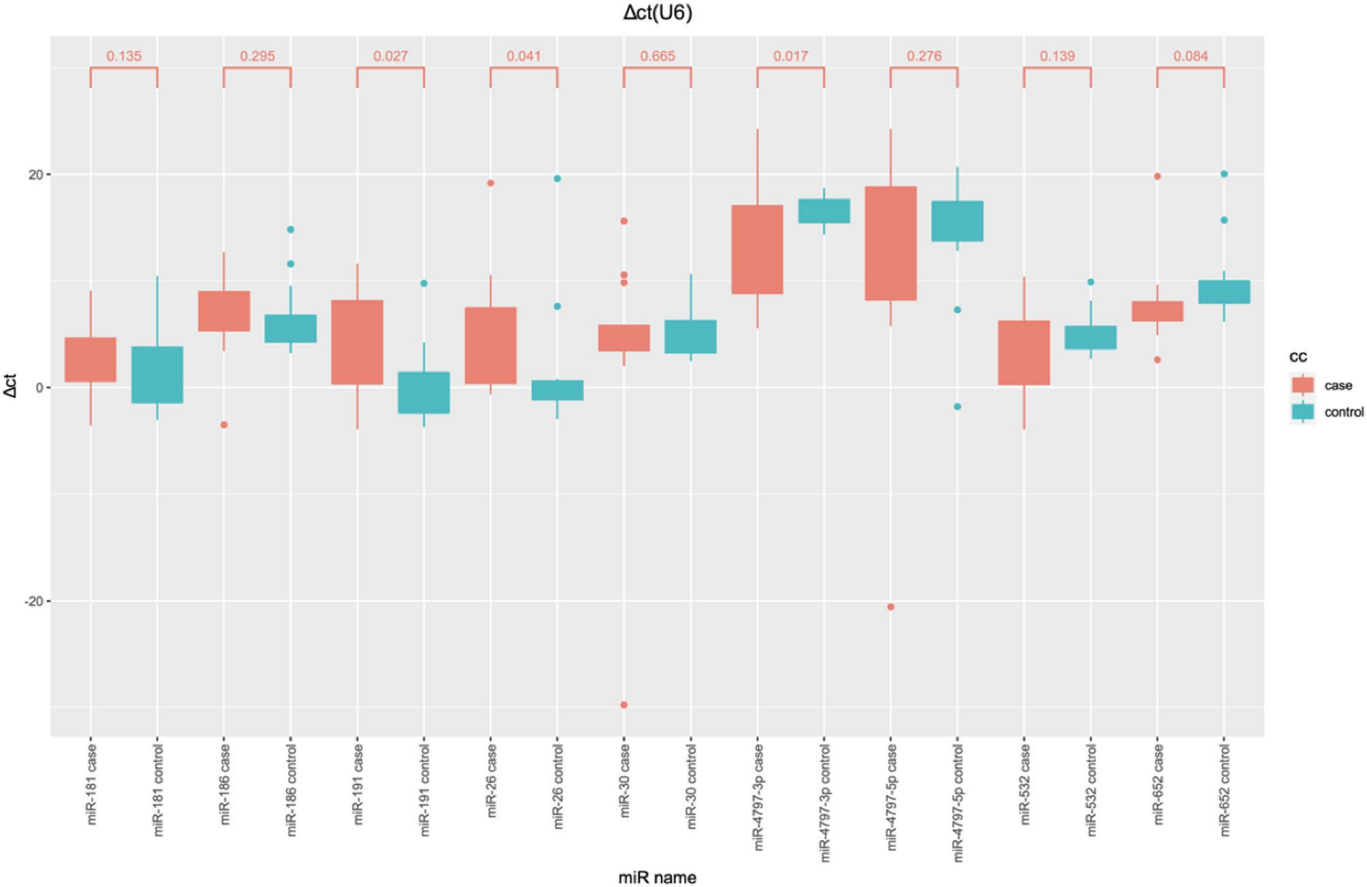
Normalization of miRs cycle threshold using U6 in the box plot. There is significant up-regulation in miR-4797-3p and miR-191 and downregulation in miR-26 in case compare to control in t-tests.

Finally, the end result of relative expression with 2^−ΔΔCT^ calculation are summarized in Table 5 and Fig. 2 presents diagram of normalized CT comparison in cases and controls. There is significant up regulation in miR-4797-3p and miR-191 (*p* value = 0.017, *p* value = 0.027) and down regulation in miR-26 (*p* value = 0.04) in case compare control in t-tests.

**Table 5:** Relative quantification of 9 miRs confirmed by RT-PCR.

Case	Age/year	Sex	hsa-miR-191-5p	hsa-miR-30e-5p	hsa-miR-4797-3p	hsa-miR-4797-5p	hsa-miR-532-5p	hsa-miR-26a-5p	hsa-miR-652-3p	hsa-miR-186-5p	hsa-miR-181a-5p	ADHD	Seizure	Autism	Genotype	CGG repeats
1	80	F	Down	Down	Up	Up	Up	Down	Up	Down	Down	Neg	Neg	Neg	Premutation	68.66/68.66
2	48	F	Down	Down	Up	Up	Up	Down	Up	Down	Down	Neg	Pos	Pos	Full mutation	312/30.2
3	44	M	Down	No diff	Up	Up	No diff	Down	Up	Down	Down	Pos	Pos	Pos	Full mutation	311.8
4	54	F	Down	Down	Up	Up	Up	Down	Up	up	Down	Neg	Neg	Neg	Premutation	104.4/104.4
5	25	M	Down	Up	Up	Down	Up	Down	Up	No diff	Down	Pos	Neg	Pos	Full mutation	310.8
6	27	M	Up	Up	Down	Down	Down	Down	no Diff	Down	Up	Pos	Pos	Pos	Full mutation	310.8
7	36	M	Up	No diff	No diff	No diff	Up	Up	Up	No diff	Down	N/A	N/A	N/A	Full mutation	312.36
8	34	M	Down	No diff	Up	Down	Down	Down	Down	Down	Down	N/A	N/A	N/A	Full mutation	319.4
9	62	F	Down	Down	Up	Down	Down	Down	up	Down	Down	N/A	N/A	N/A	Full mutation mosaic	318/22
10	29	M	UP	UP	Down	Down	UP	UP	UP	UP	Down	Pos	Neg	Pos	Full mutation	317
11	32	M	Down	Up	No diff	Down	Up	Down	Down	Down	Down	Neg	Neg	Pos	Full mutation	318
12	57	F	Down	Down	No diff	Down	Down	Down	Up	No diff	Down	Neg	Neg	Neg	Premutation	88/22
13	28	M	Down	Down	No diff	Down	No diff	Down	No diff	Down	Down	pos	Neg	Pos	Full mutation	316
14	26	M	No diff	Down	Up	Down	Down	Down	Down	No diff	Down	Neg	Neg	Pos	Full mutation	316
15	59	F	Down	No diff	No diff	Down	Down	Down	No diff	No diff	Down	Neg	Neg	Pos	Full mutation mosaic	227.2/30

*F* female, *M* male, *ADHD* attention deficit hyperactivity disorder, *N/A* not available, *no diff* no differences, *down* downregulated, *up* upregulated, *neg* negative, *pos* positive.

## Discussion

The current study identified FXS-specific changes in miRNAs among Iranian blood samples as preliminary evidence. We identified twenty-five differentially expressed miRNAs sequenced in the blood of individuals with FXS compared to the controls, and we found minor downregulation of miR-125a-5p.

The main finding of this study is that levels of three miRNAs (i.e., hsa-miR-532-5p, hsa-miR-652-3p, and hsa-miR-4797-3p) were significantly upregulated in the FXS group versus healthy controls while levels of six miRNAs (i.e., hsa-miR-191-5p, hsa-miR-181-5p, hsa-miR-26a-5p, hsa-miR-30e-5p, and hsa-miR-186-5p, hsa-miR-4797-5p) exhibited significant downregulation in patients with FXS compared to controls; and these dysregulations were confirmed by RT‐qPCR. Additionally, miR-191-5p, miR-26a-5p, and miR-181-5p downregulation have expanded priority as the focus on women as gender priority and age over 55 years.

MicroRNAs regulate mRNAs at the post-transcriptional level and therefore affect protein translation^12^. Changed miRNA expression patterns epigenetically affect almost every aspect of CNS function (i.e., in neurogenesis, synaptogenesis, and neuronal migration) and its development^33–35^. For instance, miR-532 is reliably expressed in the human brain, localized as distinct granules in distal axons and growth cones, and proposed to play a role in axon growth and guidance^36^. ZFHX3 gene, among 5 target genes of the hsa-miR-532-5p from the MiRTarBase microRNA Targets dataset, encodes a transcription factor that regulates neuronal differentiation^37^.

FXS is the first neurodevelopmental disease found to be linked to the dysfunction of the miRNA pathway^17^. The in vivo evidence of miRNA involvement in FXS pathogenesis was first provided in a study of the zebrafish model by identifying and isolating numerous miRNAs, including miR-*FMR1*-27 and miR-*FMR1*-42 in this model^38^.

Subsequent studies in the *FMR1* KO mouse models found that disruption of the regulating of miR-125a, miR-125b, and miR-132 causes early neural development and synaptic physiology^18^ and that there is an interaction between miR-34b, miR-340, and miR-148a with the Met 3′ UTR of the *FMR1* gene^39^. Moreover, by isolating mesenchymal stem cells from peripheral blood and differentiating these cells into neuronal cells, Fazeli et al.^40^ recently analyzed the expression of miR-510 by the qPCR method. The authors reported an enhanced expression of miR-510, located on chromosome X in the 27.3Xq region, flanking to a fragile X site, in the female carriers of *FMR1* full mutation.

In a most recent study, Putkonen et al.^19^ showed upregulation of miR-125a in urine from children with FXS. The investigators did not examine differential miRNA expression changes in FXS blood samples or the correlations of miR-125a levels in urine with those of in the cell-free circulation (i.e., in serum and plasma) and other body fluids^19^. In our study, we found a minor downregulation of miR-125a-5p in the blood of individuals with FXS. One preliminary hypothesis for this finding is that due to urinary secretion of miR-125a-5p, its blood level expression decreased similar to what happens for the blood-urine balance of electrolytes. In line with our results in the Alvarez-Mora MI study on FXTAS patients using deep sequencing, the authors observed a slight but not significant reduction of miR-125a-5p in the blood of FXTAS patients^41^. Recently, Frye et al. published an article on 2021 that found hsa-miR-125a-5p_R-1 was significantly down-regulated in the ASD group, which is in parallel to our current study^42^. They introduced the role of miR-181 in immunomodulation in ASD in lymphoblastoid cell lines derived from 10 individuals with ASD. They also found that out of 267 detected miRs in the ASD vs. Sibling group, 14 other miRNAs were dysregulated^42^. Furthermore, in a microarray analysis by Seno et al. the up-regulation of miR-125b as well as miR-196a, miR-650, miR-338-3p, in 20 cases of ASD were reported^43^.

Mundalil Vasu et al.^44^, found thirteen differentially expressed serum miRNAs in 55 individuals with autism spectrum disorder (ASD) compared to the controls and miR-125a was not among the dysregulated miRNAs. In their enrichment analysis, five miRNAs (miR-130a-3p, miR-19b-3p, miR-320a, miR181b-5p, and miR-572) showed a good discriminative power for distinguishing individuals with ASD.

Moreover, in concordance with our results, the association between autistic traits and X-linked SNPs in the gene family linked with FXS, is likely to be owing to a disruption in the recognition between has-miR-181 and the corresponding seed match sequences in these genes^45^; miR-181d and FMRP cooperatively regulate the axon elongation process^22, 46^. An altered expression pattern for miR-181 and miR-191 in hippocampal neuron development has been shown to occur^47^.

Even though larger studies are needed to confirm our results and investigate the effect of other miRNAs, the changes in miRNAs seen among our patients provide evidence that these miRNAs could have roles in developmental processes, nervous system homeostasis and the function of nerve cells in those with FXS.

In one recent study, 13 miRNAs were differentially expressed in maternal plasma samples from pregnant women with fetal Down syndrome versus healthy control subjects; among the others, hsa-miR-191 was upregulated and hsa-miR30e downregulated^48^. In another study, miR-26b-5p, miR-185-5p, and miR-191-5p were identified as potential biomarkers for ADHD in peripheral blood mononuclear cells^49^. Altered expression of miR-26a and miR-26b have been shown in peripheral blood of major depressive patients during antidepressant therapy, in Alzheimer's disease, and Parkinson's disease^50^. Finally, hsa-miR-532-5p and hsa-miR-652-3p are upregulated in schizophrenia^51, 52^.

Our finding demonstrates significant involvement of hsa-miR-30e-5p in FXS, which was found to be the most significantly upregulated miRNA in patients with FXS compared with controls. miR-30 family plays a major regulating role in the tissue and organ development and the pathogenesis of various clinical diseases^53^. Several studies have shown that hsa-miR-30e-5p among other miRNAs might be associated with the onset and progression of Parkinson's disease and schizophrenia^54–56^. Li et al.'s study in 2018 explained miR-30e role in MPTP-treated Parkinson's disease mice caused reduction of neuroinflammation by decreasing (nod-like receptor protein 3) Nlrp3 inflammasome activity^57^. John et al. in a review of mitochondrial MicroRNAs (mitomiRs) related to Parkinson’s disease, found miR-30e downregulation can induce mitochondrial membrane damage, free radical production, and calcium homeostasis impairment through reduced suppression of Nlrp3^58^. Similarly, Zheng et al.'s 2020 review presented additional MicroRNAs such as MiR-223, MiR-22, MiR-7, as well as MiR-30e as an inhibitor of the NLRP3 activity by targeting its UTR binding sites^59^. Sun et al. observed a significant increase in expression of has-miR-30e in both plasma samples and peripheral blood mononuclear cells (PBMC) samples amongst schizophrenia patients^60^.

Our results also showed deregulated hsa-miR-191-5p. Although, as far as we are aware, no evidence for hsa-miR-191-5p contribution to FXS has been reported so far, alterations in the expression level of hsa-miR-191-5p have earlier been found in patients with neuropsychiatric disorders sharing genetic overlap with FXS, including ASD, ADHD, schizophrenia, bipolar disorder, and major depressive disorder^49, 50, 61, 62^.

It is noteworthy that despite a large number of miRNAs associated with FXS and its related disorders that have been identified in multiple expression studies, only a few miRNAs are common between various studies. This discrepancy can be explained in part by the polygenic and complex nature of neuropsychiatric disorders^63^.

The expression profiles of the miRNAs in our study confirm some existing findings but conflict with others. For example, our result regarding the expression level of has-miR-30e in patients with FXS is consistent with Sun et al.’s^60^ report that miR-30e was upregulated in PBMCs from patients with schizophrenia. In contrast, Perkins et al.^55^ have found that miR-30e is downregulated in the prefrontal cortex of subjects with schizophrenia compared with healthy subjects. The exact reason for these conflicting results remains to be determined but it may be because of differences in screening standards (i.e., patients' ethnicity, geographical region, and screening criteria), techniques used for miRNA detection and profiling, and experimental design. Furthermore, as previously mentioned by Alvarez-Mora et al.^41^, it is documented that the expressions of miRNAs are tissue-specific and/or temporally regulated which may partially explain the differences seen between the findings of different studies.

This study is based on a small sample size and is an initial discovery on the path of diagnostic, prognostic, and treatment purposes in FXS. Several types of research to identify biomarkers of relevance to clinical trials of targeted therapeutics in FXS recommended by experts in the field^64^. Some promising efforts also help address an ongoing issue of placebo using parent-based outcome measures in the clinical trials of targeted therapeutics in FXS^65–67^.

Several limitations to our study should be addressed. First, the sample size is relatively small.

Despite this, it is among the first pieces of evidence of altered miRNA expressions in blood samples from patients with FXS. Second, although patients consisted of individuals from all across the country, they were only Iranian in origin. Third, Validation was performed on the same dataset, due to the small number of patients who were willing to take blood. Therefore, it does not provide the exact effectiveness of the miRNAs as biomarkers. It should be validated on an independent set of patients and donor specimens. Forth we examined miRNA expression changes in non-neuronal cells since neuronal tissue is not easily accessible. However, it has been shown that miRNA expression changes in the peripheral circulation are highly correlated with those of neuronal tissue from patients with various neuropsychiatric disorders^33^.

## Conclusions

Our study is among the first to present the characterization of the miRNA’s expression profiles in blood samples of patients with FXS using deep sequencing-based technologies. Out of 25 picked miRNAs, levels of nine miRNAs were found to be changed (i.e., 3 upregulated and 6 downregulated) in FXS blood. Altered peripheral miRNA levels have been identified in numerous neuropsychiatric disorders, including FXTAS, ASD, ADHD, Down syndrome, depression, and schizophrenia. Our results provide a new perspective for the role of miRNA profiling in the pathophysiology of FXS, but larger studies are required to confirm these preliminary results and explore the influence of the other dysregulated miRNAs. If confirmed, it could open the possibility of using miRNAs as novel non-invasive FXS biomarkers or broad-spectrum therapeutic agents.

## Supplementary Information

Supplementary Information 1.

Supplementary Information 2.
