# Supplementary material for: Identification of microRNAs associated with human fragile X syndrome using next-generation sequencing
Source: Sci Rep. 2022 Mar 23;12:5011. doi: 10.1038/s41598-022-08916-4 (PMC8943156; doi:10.1038/s41598-022-08916-4)
Supplement: Supplementary file 1 — Supplementary Information 1. [file 41598_2022_8916_MOESM1_ESM.pdf]

```
"" "All reads" "Mapped reads" "% mapped reads"
"MIRNA001_s1_L001_R1_001_trimmed.fastq.log" 1224062 826519 67.5226
"MIRNA002_s1_L001_R1_001_trimmed.fastq.log" 1263356 1057077 83.6721
"MIRNA003_s1_L001_R1_001_trimmed.fastq.log" 752970 473769 62.92
"MIRNA004_s1_L001_R1_001_trimmed.fastq.log" 877859 623599 71.0363
"MIRNA005_s1_L001_R1_001_trimmed.fastq.log" 811803 574546 70.774
"MIRNA006_s1_L001_R1_001_trimmed.fastq.log" 519045 430849 83.0079
"MIRNA007_s1_L001_R1_001_trimmed.fastq.log" 456453 140044 30.6809
"MIRNA008_s1_L001_R1_001_trimmed.fastq.log" 220019 157241 71.467
"MIRNA009_s1_L001_R1_001_trimmed.fastq.log" 2040335 56958 2.7916
"MIRNA010_s1_L001_R1_001_trimmed.fastq.log" 1272568 182793 14.3641
"MIRNA011_s1_L001_R1_001_trimmed.fastq.log" 784597 54728 6.9753
"MIRNA012_s1_L001_R1_001_trimmed.fastq.log" 768257 72217 9.4001
"MIRNA013_s1_L001_R1_001_trimmed.fastq.log" 966970 87946 9.095
"MIRNA014_s1_L001_R1_001_trimmed.fastq.log" 10454997 1115266 10.6673
"MIRNA015_s1_L001_R1_001_trimmed.fastq.log" 1520301 156772 10.3119
"MIRNA016_s0_L001_R1_001_trimmed.fastq.log" 1734326 172928 9.9709
"MIRNA017_s0_L001_R1_001_trimmed.fastq.log" 1223058 274973 22.4824
"MIRNA018_s0_L001_R1_001_trimmed.fastq.log" 1789524 907611 50.718
"MIRNA019_s0_L001_R1_001_trimmed.fastq.log" 1889794 785652 41.5734
"MIRNA020_s0_L001_R1_001_trimmed.fastq.log" 1345546 432990 32.1795
"MIRNA021_s0_L001_R1_001_trimmed.fastq.log" 1480491 204555 13.8167
"MIRNA022_s0_L001_R1_001_trimmed.fastq.log" 2456946 881481 35.8771
"MIRNA023_s0_L001_R1_001_trimmed.fastq.log" 1542029 643506 41.7311
"MIRNA024_s0_L001_R1_001_trimmed.fastq.log" 2164647 672595 31.0718
"MIRNA033_s0_L001_R1_001_trimmed.fastq.log" 775862 93403 12.0386
"MIRNA034_s0_L001_R1_001_trimmed.fastq.log" 1007604 164027 16.2789
"MIRNA035_s0_L001_R1_001_trimmed.fastq.log" 1272755 538443 42.3053
```

SHRiMP log table and the number of mapped reads for all miRNAs.
