# Supplementary figures and images for: Identification of microRNAs associated with human fragile X syndrome using next-generation sequencing
Source: Sci Rep. 2022 Mar 23;12:5011. doi: 10.1038/s41598-022-08916-4 (PMC8943156; doi:10.1038/s41598-022-08916-4)

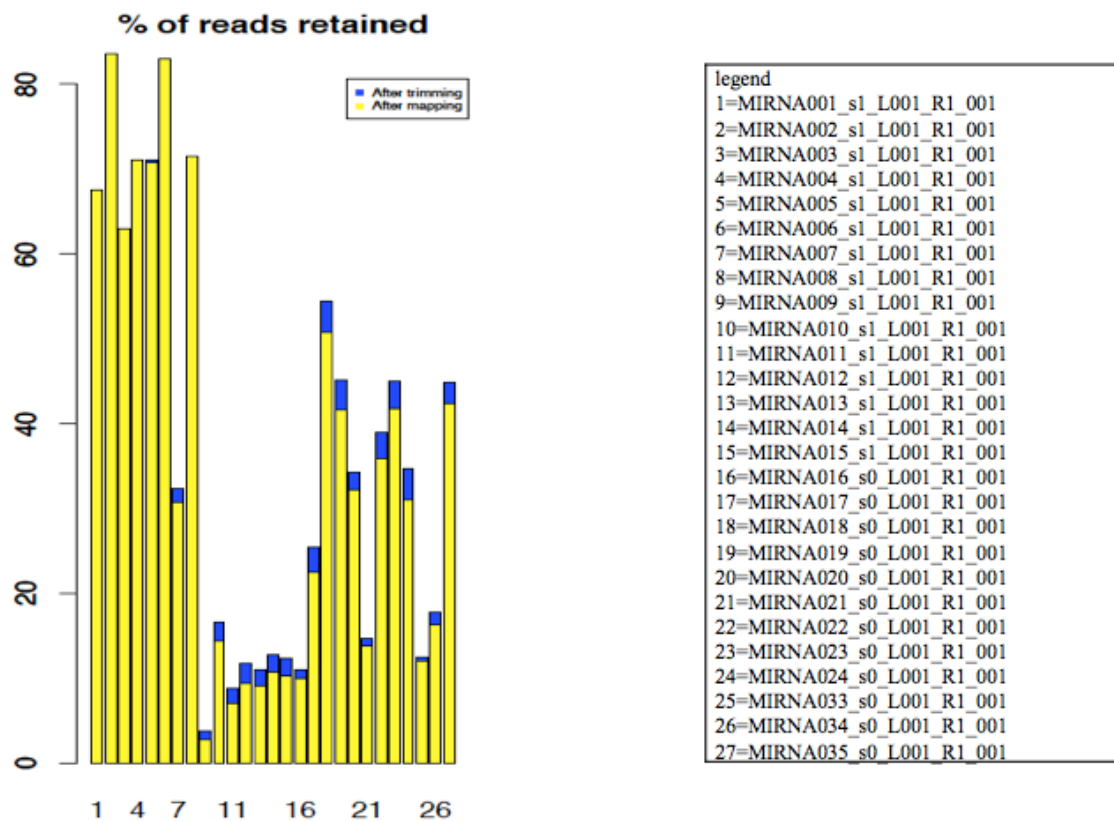

Trimming-mapping plot and the percentage of reads mapped to miRNAs in each sample

Supplement: Supplementary file 2 — Supplementary Information 2. [file 41598_2022_8916_MOESM2_ESM.pdf]
